# Questionnaire study and postmortem findings in backyard chicken flocks in Finland

**DOI:** 10.1186/s13028-015-0095-1

**Published:** 2015-01-22

**Authors:** Leena Pohjola, Laila Rossow, Anita Huovilainen, Timo Soveri, Marja-Liisa Hänninen, Maria Fredriksson-Ahomaa

**Affiliations:** Department of Production Animal Medicine, Faculty of Veterinary Medicine, University of Helsinki, Leissantie 43, FI-04920 Saarentaus, Finland; Production Animal and Wildlife Health, Finnish Food Safety Authority Evira, Mustialankatu 3, FI-00790 Helsinki, Finland; Veterinary Virology, Finnish Food Safety Authority Evira, Mustialankatu 3, FI-00790 Helsinki, Finland; Department of Food Hygiene and Environmental Health, Faculty of Veterinary Medicine, University of Helsinki, Agnes Sjöberginkatu 2, FI-00790 Helsinki, Finland

**Keywords:** Backyard poultry, Management, Postmortem, Biosecurity

## Abstract

**Background:**

Although modern commercial poultry production today is based on large farms and intensive husbandry, keeping backyard poultry has regained popularity in industrialized countries. However, the health status of backyard flocks is still relatively poorly documented. A questionnaire was sent to the owners of 376 backyard poultry flocks (<500 birds) in order to study health management procedures and characterize backyard poultry populations in Finland. Information was also collected on the postmortem findings from non-commercial flocks using necropsy data from the Finnish Food Safety Authority (Evira).

**Results:**

Backyard flocks in Finland are small in size (<50 birds), comprising mainly chickens. Based on the results of the questionnaire, the health of such flocks is good, mortality low and vaccinations are not commonly used. Most of the flocks were registered in the national poultry register. The standard biosecurity practices are not generally applied and contact with wild birds, pets and farm animals is frequent, which can make the flocks more prone to infectious diseases. We conducted an 11-year retrospective study of the postmortem necropsy findings of the Evira in order to document the diseases, which caused mortality in backyard chickens in Finland. Necropsy was performed on a total of 132 non-commercial laying hens during 2000 – 2011. The most common postmortem findings were Marek’s disease (27%) and colibacillosis (17%).

**Conclusions:**

This study is the first to report data on characteristics of and management practices for backyard chicken flocks in Finland. Close connections with commercial flocks are rare and farms are usually distantly located suggesting that the risk that these backyard flocks pose to commercial poultry is low.

## Background

Although modern commercial poultry production in industrialized countries is concentrated in large farms that practice intensive husbandry, strict biosecurity control and organized health management, raising small poultry flocks in backyards for eggs and meat has regained popularity. The health status of such backyard flocks is generally poorly documented, but interest in health and management of non-commercial flocks has increased and reports have been published from Canada [1], New Zealand [2], UK [3], and the USA [4,5].

Backyard flocks live in a close contact with wild birds and under conditions where biosecurity measures are often not implemented [6]. Therefore, the birds can be exposed to transmissible infectious diseases such as avian influenza (AI) [7,8]. Backyard flocks are known to be involved in outbreaks of avian infectious diseases and could play a role in the transmission of diseases to commercial poultry, although some epidemiological studies have indicated their role to be only marginal [9-12]. It is possible that backyard flocks could also be involved in the transmission of zoonotic diseases to humans. Newcastle disease (ND) and AI are highly contagious avian viral diseases that can infect humans. *Salmonella* and *Campylobacter*, which are common foodborne pathogens, can be transmitted from poultry and poultry products to humans [13-16]. Due to their contact with the outside environment, backyard chickens carrying pathogens can also be indicators of zoonotic enteric pathogens that circulate in wild birds and other animals.

In May 2012, there were a total of 365 small (<500 birds), non-commercial chicken flocks in the Finnish national poultry register. These backyard poultry flocks consisted mostly of native layer hen breeds, but also included other domestic gallinaceous birds such as turkeys, quails and geese. Generally, native hens are known to grow more slowly and produce fewer eggs than commercial hens. On the other hand, they are good foragers and mothers and adapt to changes in the environment. In 1998, Agrifood Research Finland (MTT) founded a program for the conservation of genetic diversity among Finnish poultry breeds. At the end of 2011, there were 285 indigenous flocks in the conservation program containing a total of 4788 chickens. Rehoming of hens from commercial farms to private premises is less common in Finland compared to UK [3].

The aim of our study was to collect data on health management procedures and characterize Finnish backyard poultry populations based on a questionnaire sent to owners of these flocks. A further aim was to collect data on the postmortem findings for non-commercial flocks using necropsy data from Evira.

## Materials and methods

In this study, backyard poultry flocks were defined as flocks where the birds were kept for eggs or other products consumed mainly by the owners and their families, and for which the overall numbers on the farm were fewer than 500 birds. A questionnaire was sent to all backyard flock owners that were registered either with the voluntary MTT chicken conservation program or the voluntary Finnish Poultry Association’s hobbyist register or both. These registers are the only voluntary registers for backyard poultry owners in Finland. The official poultry register could not be used because of the register’s privacy regulations. The questionnaires were sent between May and July 2012.

The questionnaire contained 35 questions, including both binary and open-ended questions, focused on general flock parameters, bird health, bird movement and biosecurity practices. It was possible to answer the questionnaire anonymously.

The presence of postmortem findings in non-commercial chickens was estimated through a retrospective study of results from necropsies submitted to Evira (Helsinki) during the years 2000 – 2011. The study included all dead/euthanized chickens that came from flocks <500 birds. The costs of the necropsies were met by the owners, except in 2011 when the necropsies were free during the national infectious bronchitis (IB) study.

The necropsies were performed by a poultry pathologist. Macroscopically changed tissues were further studied by histology. If there were no clear cause of death, following tissues were microscopically studied: *Bursa fabricius*, brain, lungs, heart, liver, spleen, kidneys and thigh muscle. The tissue specimens were fixed with formalin, embedded in paraffin and stained with hematoxylin and eosin (H&E). The endo- and ectoparasites were investigated from all the necropsied birds and parasites were microscopically examined and identified at species level [17].

Bacteriological examination for *Salmonella* spp. was done on liver and intestine (cecum) of all necropsied chickens by cultivation using the ISO method nr 6579:2002/Amendment 1, 2007, Annex D. Briefly, after 16–20 h pre-enrichment in BPW (Buffered Peptone Water (ISO), LAB M, Kerava, Finland) at 37°C, 100 μl of the BPW were inoculated on a selective MSRV (modified semi-solid Rappaport-Vassiliadis) medium (LAB M). After 24 h incubation, on MSRV subcultivation was performed on the selective media (XLD and Rambach). Other bacteriological/virological studies were done only if suspected on the grounds of flock history/signs and macroscopic or microscopic findings. For bacteriological cultivation, spleen, liver and the diseased tissues were sampled. Bacteriological cultivation was always performed if colibacillosis was suspected in order to ensure the diagnosis. IB virus was studied using RT-PCR [18].

## Results

Of the 378 questionnaires sent to flock owners, two were returned undeliverable and 181 were completed and returned (response rate 48%). The completed questionnaires came from all regions of Finland. Of the owners, 16 (9%) answered anonymously. Three of the questionnaires were not included in the survey: for two the flock sizes exceeded 500 birds and for another the owner answered the questionnaire twice. Finally, a total of 178 responses were used in the study.

### Flocks

All flocks included chickens and in 35% of the flocks there were at least one other gallinaceous bird, turkeys being the most common. The majority of the flocks had 11 – 50 birds (Table 1). Only nine percent of the participants had flocks of more than 50 birds. Most of the birds were kept for eggs as well as hobby/pet. Approximately 30% of the birds were also kept for meat production. Almost all birds were kept outdoors at least part of the year. About half of the farms had raised the birds at the same location for more than 5 years. Most of the owners had registered the flock on the national poultry register.

**Table 1:** **General characteristics of backyard chicken flocks in Finland**

**Characteristics**	**No. of flocks**	**%**
No. of birds in flock (n = 178)^A^		
1-10	36	20.2
11-20	65	36.5
21-50	61	34.3
51-100	9	5.1
101-200	6	3.4
201-500	1	0.6
No. of bird types represented in flock (n = 178)		
Chicken only flock	116	65.2
Two bird types in flock	34	19.1
Three bird types in flock	12	6.7
More than three bird types in flock	16	9.0
Owner-stated purpose for owning flock (n = 178)		
Eggs for family	141	79.2
Meat for family	47	26.4
Hobby, companion, pet	128	71.9
Breeding	41	23.0
House management (n = 178)		
Inside, confined to barn	4	2.2
Inside with confined outdoor access	96	53.9
Inside with free outdoor access	76	42.7
Free	2	1.1
Birds raised in the same premises		
(n = 178)		
<1 year	15	8.4
1-3 years	26	14.6
2-5 years	59	33.1
>5 years	78	43.8
Flock registered to the obligatory national		
poultry register (n = 177)		
Yes	147	83.1
No	30	16.9

^A^The total number of participants that answered this question.

### Bird movement

Bird movement was frequent: most of the participants had sold/given birds and had purchased birds during the previous 5 years (Table 2). One participant had imported birds from abroad illegally. Only 16% of the owners reported never to have purchased live birds.

**Table 2:** **Characteristics of bird movement in backyard chicken flocks in Finland**

**Characteristics**	**No. of flocks**	**%**
Birds purchased during the last 5 years (n = 177)^A^		
Yes, from Finland	145	81.9
Yes, abroad	1	0.6
No	31	17.5
How often birds are purchased (n = 174)		
Multiple times per year	13	7.5
Once a year	26	14.9
Once every 1–3 years	65	37.4
Once every 4–5 years	43	24.7
Never	27	15.5
Birds sold/given during the last 5 years (n = 178)		
Yes	135	75.8
No	43	24.2

^A^The total number of participants that answered this question.

### Biosecurity practices

Of the 178 participants, only 13% reported having used different shoes in the poultry premises and 35% had the possibility to wash their hands when leaving the premises (Table 3). On the farms, wild birds often had opportunities to be in close contact with the flock. The majority of the respondents reported that they complied with the legislation that requires keeping chickens inside during the spring migration of wild birds (March 1^st^ to May 31^st^). Most of the owners allowed visitors to visit the flock area. For almost half of the farms, the distance to the nearest known commercial poultry farm was >10 km and only few of the participants had a connection with a commercial poultry farm. Often the owners also had other farm animals and pets.

**Table 3:** **Biosecurity practices for backyard chicken flocks in Finland**

**Characteristics**	**No. of flocks**	**%**
Change shoes before/after flock contact (n = 178)^A^		
Yes	23	12.9
No	155	87.1
Possibility to wash hands in animal premises (n = 177)		
Yes	62	35.0
No	115	65.0
Wild birds have a contact with flock (n = 178)		
Yes	64	36.0
No	114	64.0
Visitors in the animal premises (n = 178)		
Often	25	14.0
Seldom	125	70.2
Never	28	15.7
Birds inside during 1.3. - 31.5. (n = 176)		
Yes	135	76.7
No	41	23.3
Connection to a commercial poultry farm (n = 178)		
Yes	10	5.6
No	168	94.4
Distance to the nearest intense poultry farm (n = 178)		
<1 km	1	0.6
1-3 km	10	5.6
4-5 km	6	3.4
6-10 km	16	9.0
>10 km	73	41.0
Don’t know	72	40.4
Other farm animals in the farm (n = 178)		
Yes	98	55.1
No	80	44.9
Pets in the farm (n = 178)		
Yes	161	90.4
No	17	9.6

^A^The total number of participants that answered this question.

### Flock health

Most of the owners reported that their flocks’ health was excellent or good (Table 4). The most common health problems reported were ectoparasites, sudden deaths and diarrhea. The mortality was low. In most flocks, mortality of adult birds was <10% during the previous year and in 38% of the flocks, there were no deaths of adult birds during the last year. Only one owner had vaccinated once the chickens (against Marek’s disease) and in one quarter of the flocks, bird(s) had been medicated during the last year. The medications were mostly routine treatments against ectoparasites (permethrin, pyrethrine) and endoparasites (fenbendazole). Of the 178 participants, 169 reported no veterinary consultation for their flock during the last year.

**Table 4:** **Owner-reported flock health and medication characteristics for backyard chicken flocks in Finland**

**Characteristics**	**No. of flocks**	**%**
Flock’s health status (n = 178)^A^		
Excellent	97	54.5
Good	73	41.0
Fair	8	4.5
Poor	0	0
Symptoms during the last 2 years (n = 178)		
No symptoms	58	32.6
Sudden death	53	29.8
Respiratory illness	15	8.4
Diarrhea	32	18.0
Ectoparasites	55	30.9
Endoparasites	9	5.1
Problems in laying	16	9.0
Neurological signs	20	11.2
Wasting	15	8.4
Tumors	9	5.1
Adult bird mortality during the last year (n = 175)		
No mortality	67	38.3
<10%	85	48.6
10-20%	14	8.0
20-30%	4	2.3
30-50%	4	2.3
>50%	1	0.6
Bird/birds medicated during the last year (n = 178)		
Yes	42	23.6
No	136	76.4
Birds vaccinated (n = 178)		
Yes	1	0.6
No	177	99.4
Bird/birds checked by a veterinarian during the		
last year (n = 178)		
Yes	9	5.1
No	169	94.9

^A^The total number of participants that answered this question.

### Postmortem findings

Necropsy was performed on a total of 132 non-commercial (<500 birds in a flock) chickens at Evira (Helsinki) during 2000 – 2011 (Table 5). The chickens examined were either spontaneously dead or euthanized by the owner. The most common postmortem findings were Marek’s disease (MD) and colibacillosis. All the chickens examined tested negative for *Salmonella* spp. One or more ectoparasite species was found from 19% of the chickens and one or more endoparasite species was found from 40% of the chickens. Eight of the chickens studied in 2011 were investigated in the case of infectious bronchitis virus and all tested were negative [18].

**Table 5:** **The postmortem (PM) findings for non-commercial laying chickens submitted to Evira Helsinki during the years 2000 – 2011**

**Year**	**No. of samples**	**No. of farms**	**PM finding (no.)**	**Endoparasites (no.)**	**Ectoparasites (no.)**
2000	2	2	Colibacillosis (1)^A^	None	None
			Tumor (non-Marek) (1)		
2001	6	4	Cannibalism (2)	*Ascaridia galli* (1)^B^	*Menacanthus stramineus* (2)^B^
			Cachexia (1)		
			Colibacillosis (1)		
			Marek’s disease (1)		
			Trauma (1)		
2002	7	5	Colibacillosis (4)	*Ascaridia galli* (2)	None
			Coccidiosis (2)	*Eimeria* spp.(2)	
			Marek’s disease (1)	*Heterakis gallinarum*(3)	
2003	9	7	Colibacillosis (3)	*Ascaridia galli (1)*	*Dermanyssus gallinae (2)*
			Marek’s disease (3)	*Capillaria* spp*. (1)*	
			Anemia (1)	*Eimeria* spp. *(2)*	
			Tumor (non-marek)(1)	*Heterakis gallinarum (1)*	
			Visceral gout (1)		
2004	20	13	Marek’s disease (8)	*Ascaridia galli (1)*	*Cnemidocoptes mutans (1)*
			Colibacillosis (3)	*Capillaria* spp*. (2)*	*Menacanthus stramineus (5)*
			*M. avium* spp. infection (2)	*Heterakis gallinarum (4)*	
			Cannibalism (2)		
			Amyloidosis (1)		
			Aspergillosis (1)		
			Cachexia (1)		
			Sepsis (1)		
			No findings (1)		
2005	4	4	Marek’s disease (2)	*Eimeria* spp*. (1)*	*Menacanthus stramineus (1)*
			Cachexia (1)		
			Coccidiosis (1)		
2006	4	4	Marek’s disease (3)	*Ascaridia galli (1)*	*Dermanyssus gallinae (2)*
			Cachexia (1)	*Capillaria* spp*. (1)*	
				*Eimeria* spp. *(1)*	*Menacanthus stramineus (2)*
				*Heterakis gallinarum (2)*	
2007	22	17	Colibacillosis (4)	*Ascaridia galli (3)*	*Cnemidocoptes mutans (3)*
			Marek’s disease (5)	*Capillaria* spp*. (1)*	*Dermanyssus gallinae (1)*
			*Clostrium. perfringens* enteritis (2)	*Eimeria* spp*. (4)*	*Menacanthus stramineus (3)*
				*Heterakis gallinarum (6)*	
			Coccidiosis (2)		
			No findings (3)		
			Aspiration pneumonia (1)		
			Cachexia (1)		
			Heart failure (1)		
			Sepsis (1)		
			Visceral gout (1)		
			Salpingoperitonitis (*P. multocida*) (1)		
2008	10	5	Cachexia (5)	*Heterakis gallinarum (6)*	*Cnemidocoptes mutans (1)*
			Colibasillosis (2)		
			Predator (2)		
			Listeriosis (1)		
2009	13	11	Marek’s disease (4)	*Capillaria* spp*. (2)*	*Cnemidocoptes mutans* (1)
			Colibacillosis (3)	*Eimeria* spp*. (2)*	*Dermanyssus gallinae* (1)
			Cachexia (2)	*Heterakis gallinarum (3)* *.*	
			Coccidiosis (1)		
			Dust pneumonia (1)		
			Heart failure (1)		
			Listeriosis (1)		
2010	13	7	Non-starter syndrome (4)	*Ascaridia galli (3)*	*Dermanyssus gallinae* (2)
			Marek’s disease (3)	*Capillaria spp. (1)*	*Menacanthus stramineus* (1)
			Coccidiosis (2)	*Eimeria spp. (3)*	
			Opaque cornea (2)	*Heterakis gallinarum (1)*	
			Colibacillosis (1)		
			Sepsis (1)		
2011	22	18	Marek’s disease (5)	*Ascaridia galli (6)*	*Dermanyssus gallinae* (1)
			Nails in gizzard (4)	*Capillaria* spp*. (4)*	
			Coccidiosis (2)	*Eimeria* spp*. (5)*	
			Heart failure (2)	*Heterakis gallinarum (3)*	
			Visceral gout (2)		
			Aspergillosis (1)		
			Cachexia (1)		
			Colibacillosis (1)		
			Dust pneumonia (1)		
			Hepatic lipidosis (1)		
			Listeriosis (1)		
			Muscular dystrophy (1)		
In total	132				

^A^The number of findings. One bird had only one finding. ^B^The number of findings. One bird could have more than one finding.

## Discussion

This study is the first to report data on characteristics of and management practices for backyard chicken flocks in Finland. Inclusion on the official national poultry register has been a legal requirement since April 2011 for non-commercial chicken owners regardless of flock size. The aim of the register is to be able to identify and trace all poultry flocks in a potential disease situation. To our surprise, the majority of the respondents (83%) reported being officially registered. The number of official registrations was expected to have been lower as the registration requirement is relatively new; however, some owners believed that MTT’s conservation register, which is a voluntary register, was the official one. It is also possible that the use of voluntary registers caused biases to our results as the owners on the voluntary registers may be more aware of the legal requirements and also be more interested in the management and diseases of chickens. The MTT conservation register is only for indigenous flocks, which also may have biased our results. But this was the only way the reach the owners.

As expected, biosecurity measures, such as footwear precautions and visitor restrictions, were uncommon among the flock owners. These results are quite similar to findings from other studies [4,19]. The possibility to wash hands on the premises was uncommon (35%). The “yes” answer would most likely have been higher if the question had been worded differently (are hands washed after flock contact?). The lack of a possibility to wash hands on the premises can raise the risk of transmitting zoonotic diseases when hands are washed later, often in the kitchen, or remain unwashed. The owners may underestimate the risk that poultry represents because salmonella cases from domestic sources in Finland are low [20,21].

According to owners’ personal opinions, flock health status was mostly good or excellent. These results can be distorted by the fact that the farms where the health situation is worse are not registered or the flock owners may have ignored the questionnaire. The most frequently reported health issues were ectoparasites (31%) followed by sudden, unexplained deaths (30%) and diarrhea (18%). The results correspond quite well with the necropsy findings in that MD and colibacillosis can cause unexplained deaths and diarrhea could result from endoparasites and colibacillosis.

It was a common practice to buy birds; 22% of owners purchased birds from another poultry farm at least once a year. Because the flock sizes are quite small, it is common that the cockerel is replaced to avoid inbreeding. Most owners (83%) purchased live birds at least once every 5 years. One of the respondents reported having purchased live birds from abroad. Associated with Finland’s excellent poultry health situation, such illegal import of live birds can pose a real infectious disease threat to Finnish poultry.

Vaccinations against infectious diseases were uncommon; only one owner had once vaccinated his chickens against MD. The owners were interested in using vaccination, but the problem was high cost and large dose requirement of the vaccines. Some of the owners had used medication; 24% of the flocks had been given some kind of medication during the last year, though most of them were prescription-free medicines. Permethrin- and pyrethrine-based sprays, which are frequently used against ectoparasites, were originally licensed for pigeons and are not allowed for use in chickens in Finland because there are no determined withdrawal periods for eggs or meat. It is problematic that there are not many licensed medicines with a determined withdrawal period that can be used at a suitable dosage to treat pet poultry.

Veterinarians visited the farms very infrequently. Only 9 of the 178 flocks had been controlled by a veterinarian during the last year. It seems that even when there were signs of disease in the flock, the owners chose not to call the veterinarian. Some of the respondents commented that there was a lack of interest and expertise of the veterinarians when it came to the backyard chickens. The owners reported that the knowledge they had about management and care of chickens was mostly acquired from other backyard poultry farmers and from backyard chicken internet sites. The infrequent consultation by veterinarians combined with limited knowledge of health and disease issues means that backyard flock owners could markedly slow down the notification of infectious diseases emerging in their flocks. It also raises the question of the welfare of these chickens if their diseases are not treated or are treated incorrectly by the owner. These results seem to be very similar to the welfare and health issues related to backyard chicken holdings in the Greater London Urban Area [3].

Connections with commercial poultry flocks were rare and distances to them were long. Only few of the owners had purchased organic chicken feed and old layers from commercial farms and the movement of birds was always unidirectional, from commercial flocks to backyard flocks. It seems that these backyard flocks do not pose a high risk of infection for commercial poultry, although the results presented here cannot be extrapolated beyond our study population.

Documented data on diseases of backyard chickens is scant because dead birds from backyard flocks are very rarely sent for postmortem examination, and because of this our results are more indicative than firm. Only 132 chickens that could be classified as backyard chickens on the basis of background information were sent for necropsy to Evira (Helsinki) between 2000 and 2011. Evira is the national, and at the same time the only, laboratory capable for full diagnostic necropsies in Finland. The background information included with the chickens was often incomplete. One evident drawback in sending the birds for necropsy seems to be its cost, which was also commented in the questionnaire. In 2011, necropsies were free during a national IB study, which moderately increased the number of necropsies. This may have caused some biases in the results. The small sample size may also bias our results as most dead backyard chickens are probably buried on the farm without any diagnostic necropsy being done on them. But to our knowledge there are very few recent studies that explore the necropsy findings for non-commercial chickens.

Marek’s disease (MD), a lymphoproliferative disease caused by a herpesvirus, was the most common necropsy finding in our material (27%). MD exists in poultry globally and failure to vaccinate leads to the persistence of MD in backyard flocks. The main postmortem findings revealed visceral and neural forms of MD and the diagnosis was based on typical macroscopic and histologic lesions such as enlargement of one or more peripheral nerves and the appearance of lymphomas. The management usually used for backyard chickens, where all age groups live in a single space, contributes to the spread of the virus, and temperature stress and limited ventilation during winter months may increase the frequency of the clinical disease [22]. Moreover, genetic factors can be important determinants of susceptibility to MD [23].

As expected, colibacillosis, and especially salpingoperitonitis, was another important cause of death in the material (17%). Colibacillosis is an infectious disease of birds caused by *Escherichia coli* and it is characterized in its acute form by septicemia, resulting in death, and in its subacute form by pericarditis, airsacculitis, salpingoperitonitis and perihepatitis [24,25]. In contrast to the acute salpingoperitonitis often seen in commercial laying hens, in backyard flocks, the infection seems to be more chronic. In older hens, the eggs are usually larger, which could cause the infection to spread more easily through the cloaca. The risk of colibacillosis increases with increasing infection pressure in the environment. In winter, when birds are housed indoors, the conditions for infection may be more favorable. Also it is notable that in spring 2011, infectious bronchitis virus (IBV) was found for the first time from backyard farms in Finland [17]. IBV infection is frequently associated with secondary infections such as *E. coli* that can lead to increased morbidity and mortality [26]. Eight of the chickens necropsied in 2011 were investigated in the case of IBV, but all tested were negative. Other virological studies were not done because there were no suspicious signs or macroscopic/microscopic findings. This, of course, does not prove that these backyard chickens are totally free of virus diseases such as infectious bursal disease (IBD) or IB, but it indicates that these diseases were not the cause of the death in necropsied chickens.

Our results are similar to those for a 5-year retrospective study done in northern California, where MD was the most common viral disease (22%) and *E. coli* was the most common bacterial infection (7.5%) causing mortality among backyard chickens [27].

## Conclusions

Backyard poultry flocks in Finland usually comprise <50 birds. The owner-reported health status of the flocks is good, mortality low and vaccinations are not commonly used. Adequate biosecurity practices are usually not applied and therefore contact with wild birds and other pets/farm animals can be frequent, which increases the susceptibility of the chickens to infectious diseases. These birds live in close contact with their owners and the risk for transmission of zoonotic enteric pathogens, such as *Salmonella* and *Campylobacter*, from birds to other animals and to humans exists. Connections with commercial flocks were rare and farms were distantly located, suggesting that the risk that these backyard flocks pose to commercial poultry is low.

## References

[1] Burns TE, Kelton D, Ribble C, Stephen C (2011). Preliminary investigation of bird and human movements and disease-management practices in noncommercial poultry flocks in southwestern British Columbia. Avian Dis.

[2] Zheng T, Adlam B, Rawdon TG, Stanislawek WL, Cork SC, Hope V (2011). A cross-sectional survey of influenza A infection and management practices in small rural backyard poultry flocks in New Zealand. New Zealand Vet J.

[3] Karabozhilova I, Wieland B, Alonso S, Salonen L, Häsler B (2012). Backyard chicken keeping in the Greater London Urban Area: welfare status, biosecurity and disease control issues. Br Poult Sci.

[4] Smith EI, Reif JS, Hill AE, Slota KE, Miller RS, Bjork KE (2012). Epidemiologic characterization of Colorado backyard bird flocks. Avian Dis.

[5] Beam A, Garber L, Sakugawa J, Kopral C (2013). Salmonella awareness and related management practices in U.S. urban backyard chicken flocks. Prev Vet Med.

[6] Conan A, Goutard FL, Sorn S, Vong S (2012). Biosecurity measures for backyard poultry in developing countries: a systematic review. BMC Vet Res.

[7] McBride MD, Hird DW, Carpenter TE, Snipes KP, Danaye-Elmi C, Utterback WW (1991). Health survey of backyard poultry and other avian species located within one mile of commercial California meat-turkey flocks. Avian Dis.

[8] Terregino C, De Nardi R, Guberti V, Scremin M, Raffini E, Martin AM (2007). Active surveillance for avian influenza viruses in wild birds and backyard flocks in Northern Italy during 2004 to 2006. Avian Pathol.

[9] Tiensin T, Nielen M, Songserm T, Kalpravidh W, Chaitaweesub P, Amonsin A (2007). Geographic and temporal distribution of highly pathogenic avian influenza A virus (H5N1) in Thailand, 2004–2005: an overview. Avian Dis.

[10] Akey BL (2003). Low-pathogenic H7N2 avian influenza outbreak in Virginia during 2002. Avian Dis.

[11] Capua I, Dalla PM, Mutinelli F, Marangon S, Terregino C (2002). Newcastle disease outbreaks in Italy during 2000. Vet Rec.

[12] Bavinck VA, Bouma A, van Boven M, Bos ME, Stassen E, Stegeman JA (2009). The role of backyard poultry flocks in the epidemic of highly pathogenic avian influenza virus (H7N7) in the Netherlands in 2003. Prev Vet Med.

[13] Anderson AS, Bauer H, Nelson CB (1955). Salmonellosis due to Salmonella typhimurium with Easter chicks as likely source. J Am Med Assoc.

[14] Friedman CR, Neimann J, Wegener HC, Tauxe RV (2000). Epidemiology of *Campylobacter jejuni* infections in the United States and other industrialized nations. Campylobacter. Volume II/6.

[15] Iqbal M (2009). Controlling avian influenza infections: The challenge of the backyard poultry. J Mol Gen Med.

[16] Loharikar A, Briere E, Schwensohn C, Weninger S, Wagendorf J, Scheftel J (2013). Four multistate outbreaks of human Salmonella infections associated with live poultry contact, United States, 2009. Zoonoses Public Health.

[17] Foreyt WJ (2001). Veterinary Parasitology: Reference Manual.

[18] Pohjola LK, Ek-Kommonen SC, Tammiranta NE, Kaukonen ES, Rossow LM, Huovilainen TA (2014). Emergence of avian infectious bronchitis in a non-vaccinating country. Avian Pathol.

[19] Garber L, Hill G, Rodriguez J, Gregory G, Voelker L (2007). Non-commercial poultry industries: surveys of backyard and gamefowl breeder flocks in the United States. Prev Vet Med.

[20] Anonymous (2010). European Food Safety Authority. Analysis of the baseline survey on the prevalence of *Campylobacter* in broiler batches and of *Campylobacter* and *Salmonella* on broiler carcasses in the EU, 2008 - Part A: *Campylobacter* and *Salmonella* prevalence estimates. EFSA J.

[21] Lievonen S, Ranta J, Maijala R. Salmonella in egg production in Finland - a quantitative risk assessment. National Veterinary and Food Research Institute EELA Publications; 2006. http://www.evira.fi/files/attachments/en/risk_assessment/kananmunasalmonella_sisus.pdf.

[22] Schat KA, Nair K, Saif YM, Fadly AM, Glisson JR, McDougald LR, Nolan LK, Swayne DE (2008). Marek’s disease. Diseases of Poultry.

[23] Bacon LD, Hunt HD, Cheng HH (2001). Genetic resistance to Marek’s disease. Curr Top Microbiol Immunol.

[24] Lutful Kabir SM (2010). Avian colibacillosis and salmonellosis: a closer look at epidemiology, pathogenesis, diagnosis, control and public health concerns. Int J Environ Res Public Health.

[25] Barnes HJ, Nolan LK, Vaillancourt JP, Saif YM, Fadly AM, Glisson JR, McDougald LR, Nolan LK, Swayne DE (2008). Colibacillosis. Diseases of Poultry.

[26] Cavanagh D, Gelb J, Saif YM, Fadly AM, Glisson JR, McDougald LR, Nolan LK, Swayne DE (2008). Infectious bronchitis. Diseases of Poultry.

[27] Mete A, Giannitti F, Barr B, Woods L, Anderson M (2013). Causes of mortality in backyard chickens in Northern California: 2007–2011. Avian Dis.

